# A structural vulnerability model of autism derived from symptom-ability network analysis

**DOI:** 10.3389/fpsyt.2026.1837552

**Published:** 2026-10-19

**Authors:** Hao-Ming Li, Zhimin Chen

**Affiliations:** 1 Department of Clinical Psychology, Wenzhou Seventh People’s Hospital, Wenzhou, China; 2 Department of Pediatrics, Wenzhou Central Hospital, Wenzhou, China

**Keywords:** autism spectrum disorder, gender differences, network analysis, receptive language, structural vulnerability

## Abstract

**Background:**

While Autism Spectrum Disorder (ASD) is increasingly understood through a developmental systems lens, the structural properties that confer vulnerability remain poorly defined. This study aimed to formalize and test a “Structural Vulnerability Model” by comparing the symptom-ability network architectures of preschool children with and without ASD.

**Methods:**

Utilizing a large dataset (N = 1,502 ASD; N = 570 Typically Developing [TD]), we constructed Gaussian Graphical Models and Directed Acyclic Graphs (DAGs) from ten variables spanning core symptoms, adaptive behaviors, and cognitive abilities. We performed a multi-level comparison of network topology, node centrality, gender-specific patterns, and conditional-dependence structures.

**Results:**

The ASD and TD networks differed in global strength and in the pattern of prominent edges. Communication (COM) had the highest strength centrality in TD, whereas Receptive Language (RL) had the highest strength in ASD. Exploratory bridge-centrality analyses identified RL and COM as prominent cross-domain connectors in ASD. The cross-sectional DAG analysis summarized conditional-dependence structures and was not interpreted causally.

**Conclusion:**

The findings provide preliminary, network-based support for a Structural Vulnerability Model, interpreted as a pattern of concentrated associations rather than a validated causal or prognostic mechanism. Longitudinal, externally replicated, and covariate-adjusted studies are needed before intervention implications can be drawn.

## 1 Introduction

Autism Spectrum Disorder (ASD) presents a profound developmental paradox (1). Defined by a core dyad of social communication deficits and restricted, repetitive behaviors (2), the diagnosis belies the vast heterogeneity in clinical presentation and long-term outcomes. Why do some children acquire language with ease while others remain nonverbal? Why do two children with similar early profiles follow dramatically different developmental trajectories (3, 4)? A growing consensus suggests the answers lie not in a simple list of deficits, but in the very architecture of the developing mind (3). This systems perspective reframes ASD as a disorder of dysregulation, where the dynamic interplay between cognitive, linguistic, social, and motor domains (5) shapes developmental pathways. To decipher this complex architecture, we must move beyond studying abilities in isolation and instead map the intricate web of connections that defines the system’s structure, resilience, and points of failure (6).

Network analysis offers a powerful methodology to construct such a map, conceptualizing abilities as nodes and their relationships as edges (7, 8). This approach can reveal the system’s topology, yet its application to ASD has raised more questions than it has answered. A particularly compelling parallel exists with neuroscience, where the “local over-connectivity and long-range under-connectivity” theory posits that ASD brains are characterized by excessively strong connections within local regions but weak connections between distant functional hubs (9, 10). This neural blueprint suggests a system that is functionally fragmented and inefficient. A critical, unanswered question is whether this neural pattern has a behavioral and psychological counterpart. Do children with ASD exhibit a developmental architecture of “Isolated Modules”—domains of ability that are rigidly over-coupled internally but poorly integrated with one another? While prior network studies have noted structural differences between ASD and typically developing (TD) groups (11), they have not yet articulated a comprehensive model of why these differences confer vulnerability.

Prior network research has highlighted heterogeneous network organization in autism and related developmental phenotypes, and network approaches have been increasingly applied to chart how symptom organization changes across child development (12). Our study builds on this literature by focusing specifically on the joint organization of core symptoms, adaptive functioning, and cognitive abilities in preschool children.

This structural question brings a second, more specific puzzle into sharp focus: the role of language. Decades of longitudinal research have consistently identified early receptive language (RL) as one of the most potent predictors of long-term social, cognitive, and academic outcomes in ASD (13, 14). Yet, its status as a predictor is a statistical observation, not a mechanistic explanation. The present network analysis therefore examines whether RL occupies a structurally prominent position in the observed cross-sectional symptom-ability configuration, without inferring a developmental mechanism.

Finally, a complete structural model must account for the well-documented heterogeneity in ASD, particularly concerning sex and developmental profiles (15–18). Phenotypically, girls with ASD often present differently from boys, a finding that may be linked to the female protective effect, which suggests a higher etiological threshold for females. We therefore examine whether the estimated cross-sectional network configurations differ descriptively by sex, while recognizing that such comparisons cannot establish sex-specific developmental processes. Developmental cascade frameworks provide a useful longitudinal perspective (19); however, the present data cannot identify temporal ordering or the direction of developmental effects.

To address these interconnected puzzles, we propose and test a Structural Vulnerability Model of the ASD network. We hypothesize that the TD and ASD groups will show different patterns of estimated conditional associations and central nodes. We also explore whether these descriptive configurations differ between boys and girls. Finally, the DAG analysis is used to summarize alternative oriented representations of the observed conditional-dependence structure; it is not used to test bottom-up or top-down developmental causation.

## 2 Methods

### 2.1 Participants and data source

This study conducted a secondary analysis of a publicly available dataset originally collected as part of the Get SET Early program (20). The data are accessible via the authors’ public GitHub repository. In the original study, informed consent was obtained from the legal guardians of all participants, and the research protocol was approved by the University of California, San Diego’s ethics committee.

Our analysis focused on a subset of the cross-sectional data from the initial visit. The original dataset contained 1,539 children with ASD and 601 TD children (20). After applying a complete case analysis to handle missing values for the variables in our study, the final analytical sample for the present research included 1,502 preschool-aged children diagnosed with ASD (1,171 boys, 331 girls; approximate male-to-female ratio of 3.5:1) and 570 TD preschool-aged children (332 boys, 238 girls). The mean age of the ASD group was 26.2 months (range: 12–47 months), and the mean age of the TD group was 25.2 months (range: 12–48 months).

### 2.2 Measures and variables

Ten variables were extracted from three standardized assessments to construct the network nodes: the Autism Diagnostic Observation Schedule (ADOS; Social Affect [SA] and Restricted and Repetitive Behaviors [RRB]) (21), the Vineland Adaptive Behavior Scales (Vineland; Communication [COM], Socialization [SOC], Daily Living Skills [DLY], and Motor Skills [MTR]) (22), and the Mullen Scales of Early Learning (MSEL; Fine Motor [FM], Visual Reception [VR], Receptive Language [RL], and Expressive Language [EL]) (23).

### 2.3 Data analysis

Network structures were estimated within a Gaussian Graphical Model (GGM) framework utilizing the EBICglasso method to regularize the networks and determine the optimal sparsity (24). The EBICglasso tuning parameter was gamma = 0.5, corresponding to the default setting used by the bootnet EBICglasso estimator. Node importance was evaluated using strength, betweenness, closeness, and expected influence; strength was the prespecified primary centrality index (25). Network stability and edge-weight accuracy were assessed using case-dropping and non-parametric bootstrapping (1,000 resamples), evaluating centrality stability via CS-coefficients (26). Subgroup network differences in global strength and individual edges were evaluated using the Network Comparison Test (NCT; 1,000 permutations) (27). Directed acyclic graphs (DAGs) were estimated using score-based hill-climbing with BIC-g scoring and 1,000 bootstrap resamples, retaining edges with at least 85% strength confidence (28, 29). All analyses were performed in R using the bootnet, qgraph, NetworkComparisonTest, and bnlearn packages.

Structural vulnerability was treated as an interpretive network descriptor rather than a single scalar index. It refers to the joint pattern of global strength, prominent within-domain edges, and node-level centrality. Global strength is the sum of absolute edge weights and is not a measure of network density. We conducted exploratory bridge-centrality analyses using the *a priori* assessment domains (ADOS, Vineland, and MSEL), following prior applications of bridge centrality in clinical populations (30); raw bridge statistics were used to identify bridging nodes, not as a formal between-group test of inter-domain integration. Edge-level NCT permutation p values were not multiplicity-adjusted and are reported only as exploratory. Age was available but was not included because the network was restricted to the selected symptom and ability indicators. Developmental quotient was not separately modeled because it would be a composite overlapping with MSEL component nodes. DAG directions were not interpreted as temporal, developmental, or causal effects, nor as unique orientations within a Markov equivalence class.

For exploratory sex comparisons, complete-case boys and girls were compared using two-sided independent-samples Student’s t tests. Cohen’s d was calculated using the pooled SD (male minus female; positive values indicate higher male scores). The 10 within-group sex-comparison p values were adjusted using the Benjamini-Hochberg false-discovery-rate procedure, and the resulting q values are reported in **Tables 1**, **2**.

**Table 1:** Descriptive statistics and gender difference tests for study variables (ASD group).

Variable	ASD(N = 1502)		Male (n=1171)		Female (n=331)		t	Cohen’s d	q (FDR)
	Mean	SD	Mean	SD	Mean	SD			
SA	13.84	4.85	13.72	4.86	14.25	4.81	−1.779	−0.111	0.3668
RRB	4.71	2.21	4.74	2.22	4.62	2.16	0.846	0.053	0.5249
COM	74.53	15.11	74.36	15.21	75.12	14.76	−0.807	−0.050	0.5249
SOC	82.64	12.28	82.54	12.37	82.98	12.00	−0.567	−0.035	0.5709
DLY	82.91	13.10	82.50	13.24	84.35	12.48	−2.279	−0.142	0.2280
MTR	90.48	11.06	90.68	11.11	89.76	10.84	1.331	0.083	0.3668
FM	81.37	18.77	81.02	18.73	82.63	18.89	−1.383	−0.086	0.3668
VR	77.30	21.70	77.71	22.02	75.82	20.53	1.398	0.087	0.3668
RL	52.97	28.80	53.25	28.39	51.97	30.22	0.715	0.044	0.5276
EL	53.72	24.27	53.31	24.10	55.15	24.82	−1.216	−0.076	0.3738

Values are mean (SD). Sex comparisons used two-sided independent-samples Student’s t tests (df = 1,500). q values are Benjamini-Hochberg FDR-adjusted across the 10 within-group comparisons. Cohen’s d (male minus female; pooled SD) is reported for each variable; positive values indicate higher male scores. *q <.05.

**Table 2:** Descriptive statistics and gender difference tests for study variables (TD group).

Variable	TD(N = 570)		Male (N = 332)		Female (N = 238)		t	Cohen’s d	q (FDR)
	Mean	SD	Mean	SD	Mean	SD			
SA	3.09	2.67	3.36	2.87	2.72	2.32	2.862	0.243	0.0109*
RRB	0.61	1.00	0.73	1.12	0.44	0.80	3.418	0.290	0.0034*
COM	99.29	10.64	98.25	10.03	100.73	11.29	−2.762	−0.235	0.0118*
SOC	103.42	8.83	102.72	8.80	104.39	8.80	−2.241	−0.190	0.0357*
DLY	99.20	10.93	97.94	10.65	100.95	11.08	−3.279	−0.279	0.0037*
MTR	99.41	9.12	99.48	8.90	99.31	9.44	0.226	0.019	0.8213
FM	109.32	20.86	108.80	24.08	110.05	15.30	−0.705	−0.060	0.5348
VR	109.77	16.70	108.24	16.30	111.90	17.06	−2.591	−0.220	0.0164*
RL	103.65	21.56	101.05	19.96	107.28	23.16	−3.434	−0.292	0.0034*
EL	96.51	18.78	95.06	17.74	98.55	20.02	−2.195	−0.186	0.0357*

Values are mean (SD). Sex comparisons used two-sided independent-samples Student’s t tests (df = 568). q values are Benjamini-Hochberg FDR-adjusted across the 10 within-group comparisons. Cohen’s d (male minus female; pooled SD) is reported for each variable; positive values indicate higher male scores. *q <.05.

## 3 Results

### 3.1 Masking of typical gender-related developmental differences in ASD

Prior to network analysis, we conducted descriptive statistics and exploratory sex comparisons for all study variables (**Tables 1**, **2**). After Benjamini-Hochberg false-discovery-rate correction across the 10 within-group comparisons, no ASD variable differed by sex (all q >= .228; Cohen’s d range, -.142 to.087). In the TD group, eight variables differed by sex after correction (all q <= .036; Cohen’s d range, -.292 to.290): girls scored higher on COM, SOC, DLY, VR, RL, and EL, whereas boys scored higher on SA and RRB. These results are descriptive and do not establish sex-specific developmental processes.

### 3.2 Comparison of TD and ASD network structures

The ASD and TD networks differed in global strength and in the pattern of prominent edges (**Figure 1**). We use the term structural vulnerability as an interpretive descriptor of this configuration, rather than as a validated single metric or a synonym for network density.

**Figure 1:**
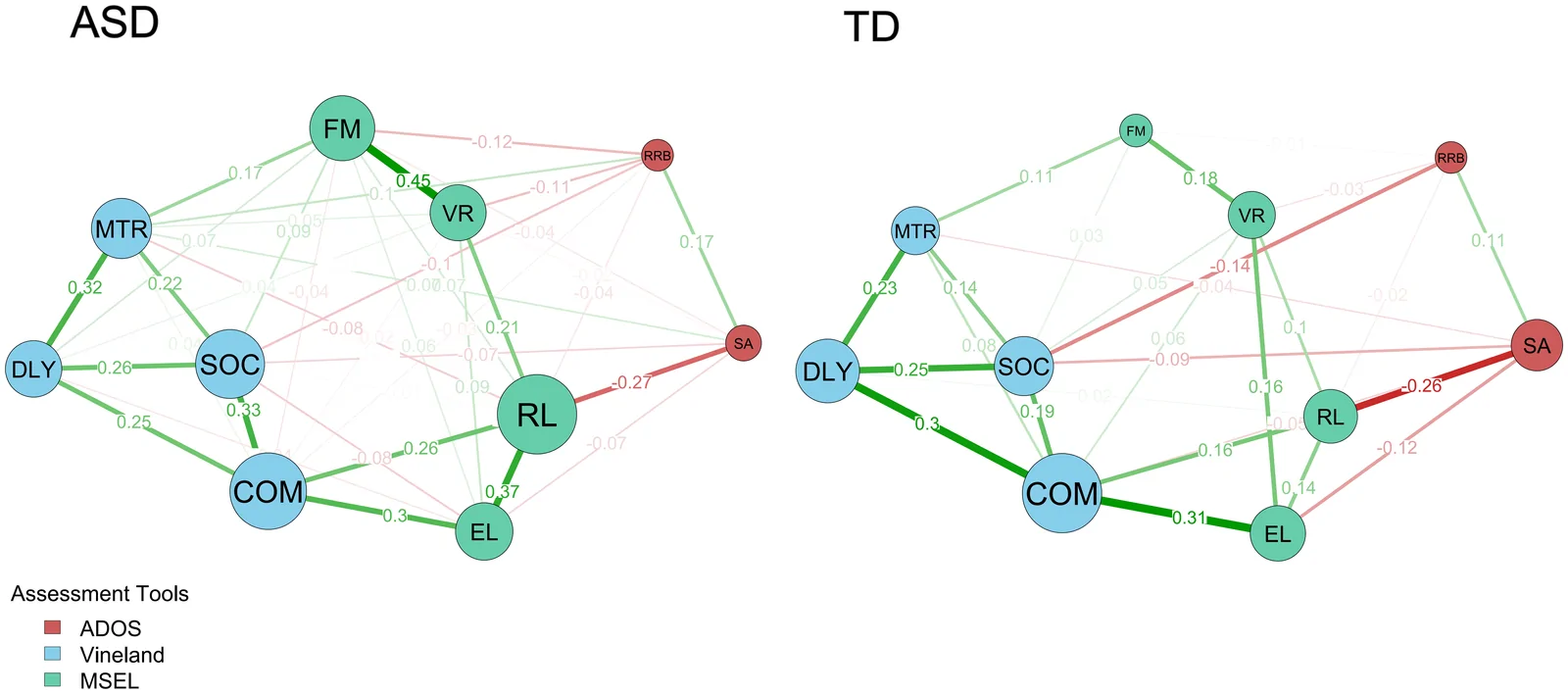
Comparison of symptom-ability network structures between ASD and TD groups.

In the TD network, adaptive Communication (COM) showed strong associations with Expressive Language (EL; weight = 0.31) and Daily Living Skills (DLY; weight = 0.30). These associations describe the estimated conditional-dependence structure and do not by themselves demonstrate compensatory processes.

In the ASD network, the largest estimated edges included Fine Motor and Visual Reception (FM-VR; weight = 0.45) and Receptive and Expressive Language (RL-EL; weight = 0.37). This concentration of prominent associations within selected ability pairs motivates the structural-vulnerability hypothesis. However, because no normalized between-community integration statistic was estimated, the present data do not provide a formal group-level test of reduced inter-domain integration.

The ASD network also exhibited a negative FM-RRB edge (weight = -0.12), whereas the corresponding TD estimate was small (weight = -0.01). This difference is descriptive and should not be interpreted as a causal association.

### 3.3 Core nodes of the vulnerable ASD network

Strength centrality was highest for RL in the ASD network. In contrast, COM had the highest strength in the TD network, with other adaptive nodes also contributing (**Figure 2**). The NCT indicated higher global strength in ASD than TD (difference = 1.79, p <.001). Because global strength summarizes absolute edge weights, it should not be described as network density. Individual edge-level NCT p values were unadjusted; therefore, we do not use them as confirmatory evidence for specific edge differences.

**Figure 2:**
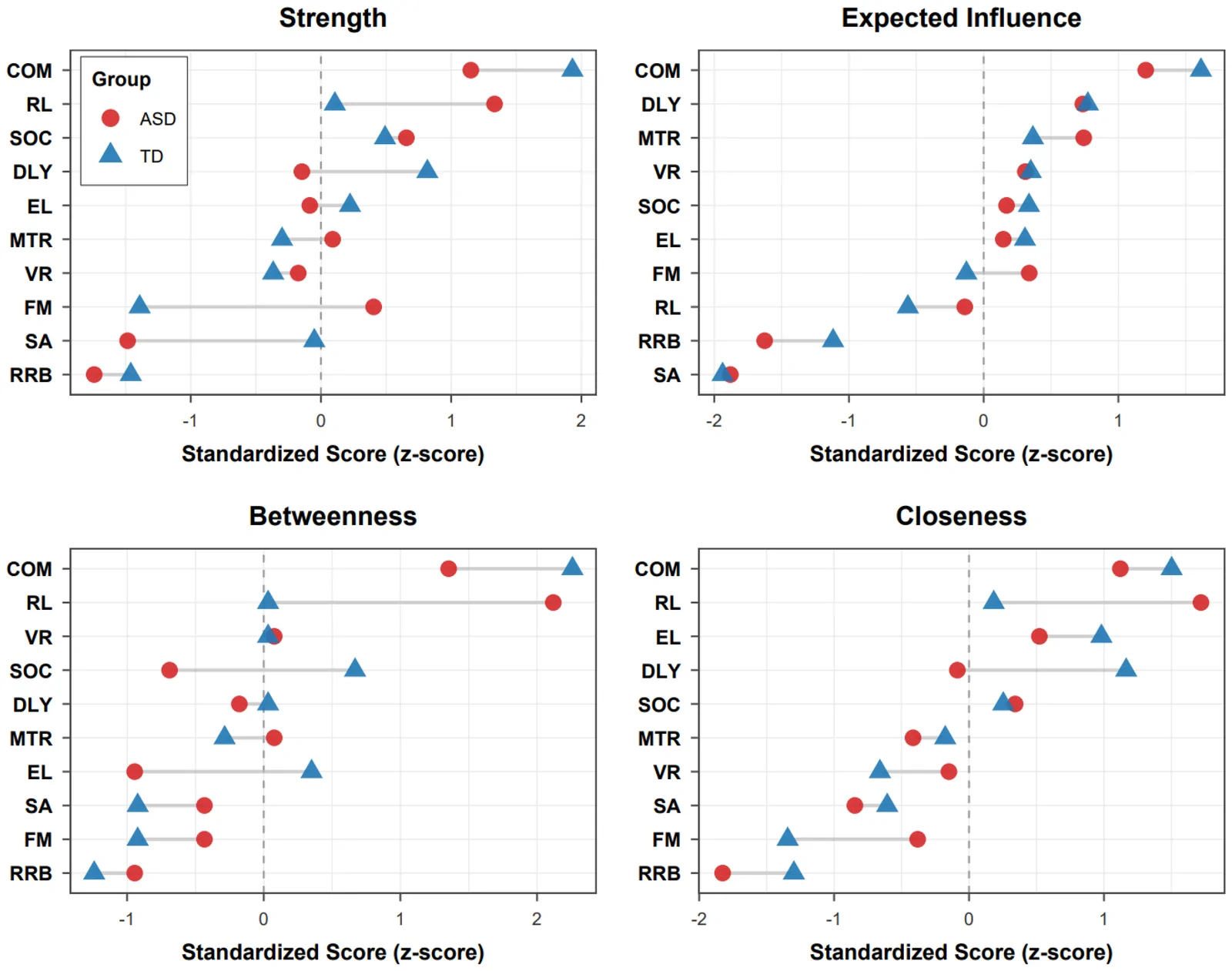
Comparison of network centrality indices between ASD and TD groups.

In exploratory bridge-centrality analyses defined by assessment domain, RL (bridge strength = 0.661) and COM (0.655) were the highest bridge-strength nodes in ASD. In TD, COM (0.584), SA (0.553), and RL (0.465) had the highest bridge strengths. These raw values identify candidate cross-domain connectors, but because they are affected by overall connection strength and were not compared with a normalized inferential test, they do not establish lower inter-domain integration in ASD.

### 3.4 Stability of centrality indices

Within the submitted EBICglasso specification, case-dropping bootstrap analyses indicated good stability for ASD strength, closeness, and betweenness (CS = 0.75 for each). In TD, strength (CS = 0.672) and closeness (CS = 0.595) also exceeded the conventional minimum stability threshold. RL had the highest strength centrality in the ASD network. These results support stability within this specification and resampling procedure; they do not establish robustness across alternative network estimators, tuning parameters, or node sets, which were not examined.

### 3.5 Different core nodes within a shared vulnerability pattern

The gender-stratified analyses were exploratory (**Figure 3**). They were intended to describe potentially heterogeneous network patterns, not to establish sex-specific effects.

**Figure 3:**
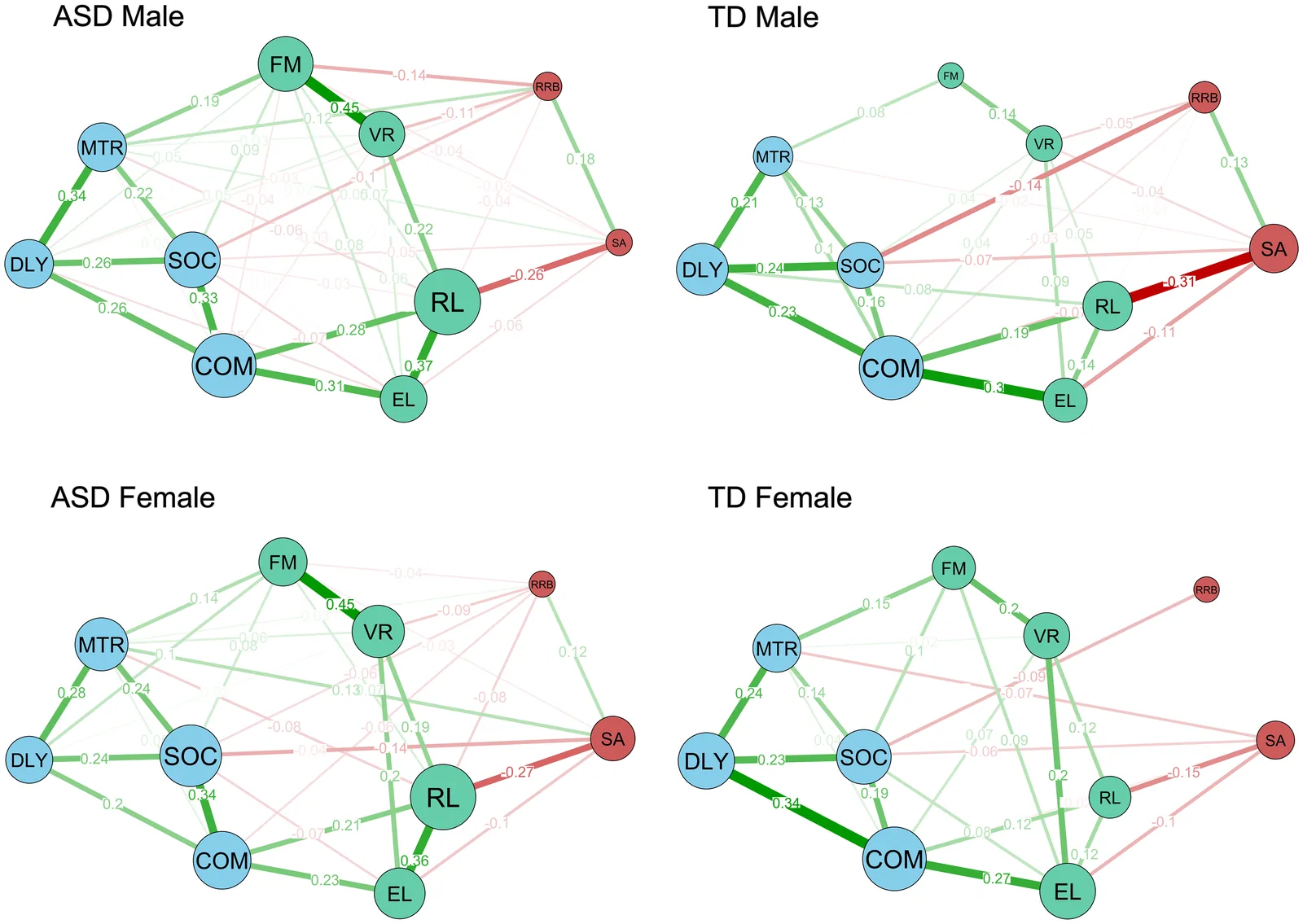
Gender-stratified network structures for ASD and TD subgroups.

In the male ASD subsample, the FM-VR (ASD: 0.45 vs. TD: 0.14) and RL-EL (ASD: 0.37 vs. TD: 0.14) edges were larger than the corresponding estimates in TD boys. These descriptive differences are consistent with the overall pattern of concentrated associations but do not demonstrate a distinct developmental mechanism.

In the female ASD subsample, some estimated within-domain edges were also larger than those in TD girls, and SA showed associations with adaptive nodes. However, the ASD female sample was substantially smaller than the ASD male sample (331 vs. 1,171). The female findings should therefore be considered exploratory, especially because no sample-size-balanced resampling sensitivity analysis was conducted.

Sex-stratified NCTs showed higher global strength for ASD than TD in both male (difference = 2.03, p <.001) and female (difference = 1.56, p <.001) comparisons (**Figure 4**). These results do not eliminate the possibility that unequal subgroup sizes influenced precision, and they should not be interpreted as confirming a universal sex-invariant pattern.

**Figure 4:**
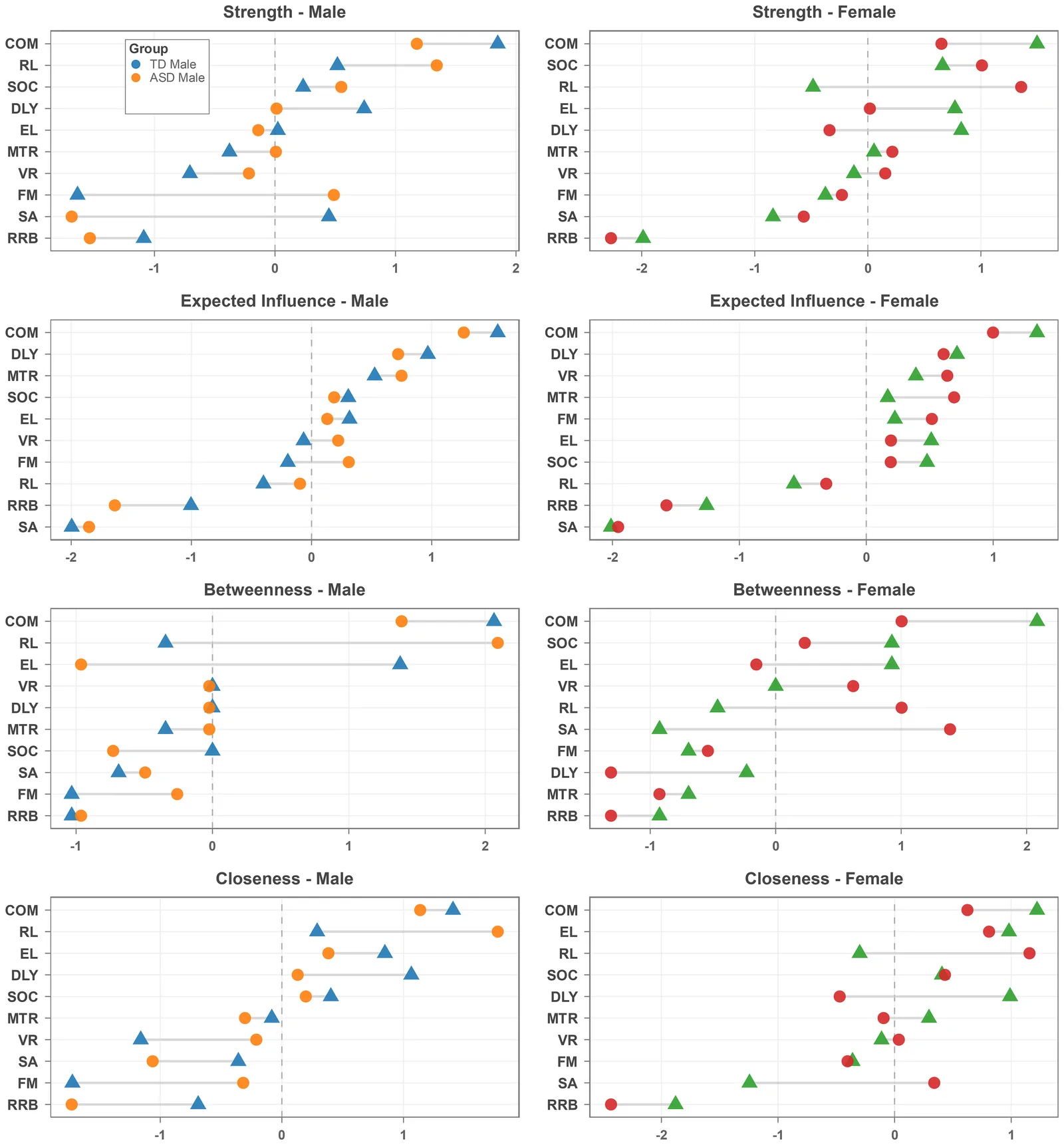
Comparison of centrality indices across gender subgroups.

### 3.6 DAG analysis of conditional-dependence structures

To summarize conditional-dependence structures, we constructed Bayesian networks (**Figure 5**). In the TD group, COM was connected to DLY, SOC, RL, and EL in the estimated DAG. These oriented edges are algorithmic representations of the fitted model, not evidence of temporal ordering, developmental direction, or causal influence.

**Figure 5:**
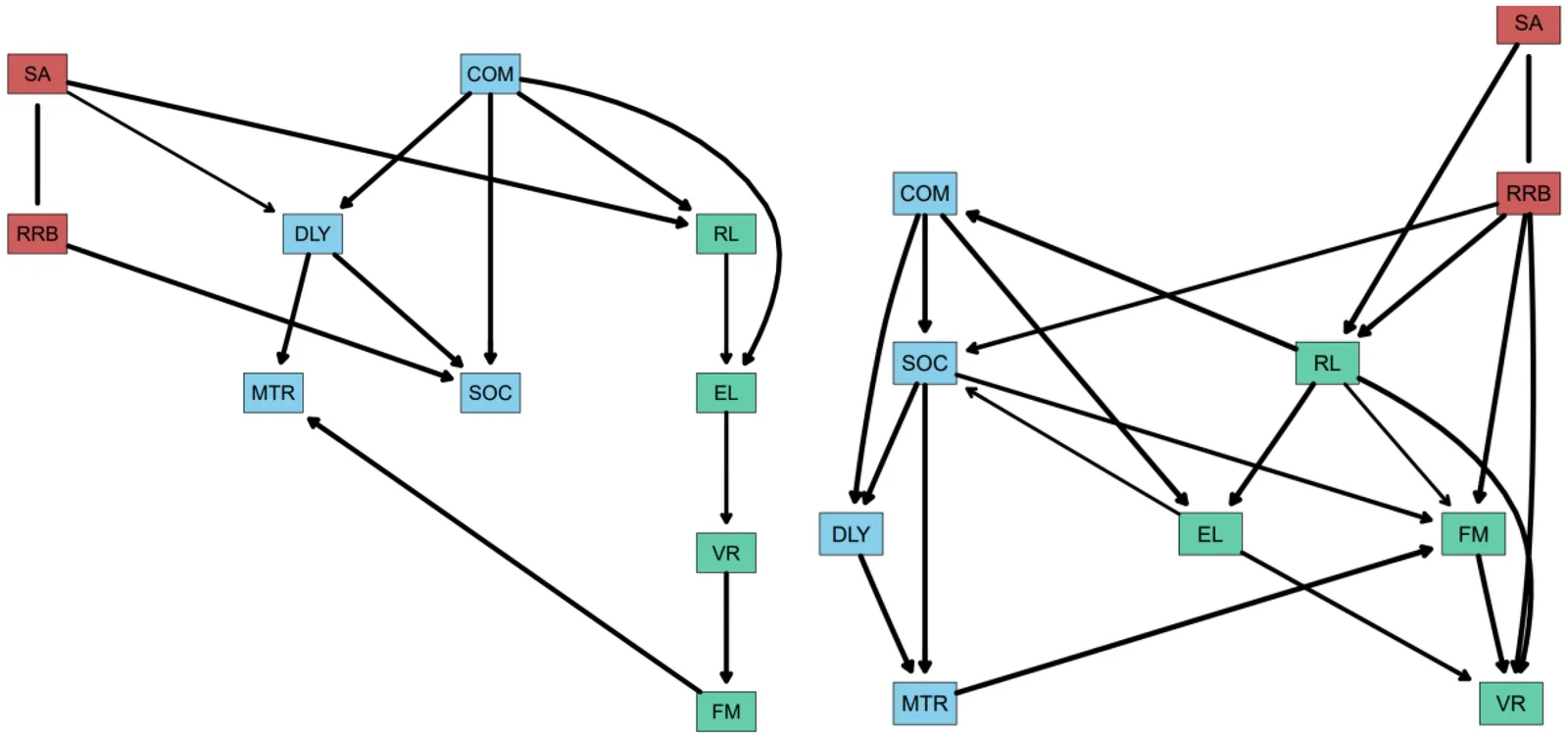
Directed acyclic graph (DAG) analysis of conditional-dependence structures.

In the ASD DAG, SA and RRB were connected to RL within the fitted structure. We interpret this pattern only as a conditional-dependence hypothesis. With cross-sectional data, statistically equivalent DAGs may encode the same distribution; consequently, the arrow directions cannot distinguish developmental causation from alternative equivalent orientations.

## 4 Discussion

Our study proposes a Structural Vulnerability Model as a hypothesis-generating description of symptom-ability network organization in ASD. The observed configuration included higher global strength and several prominent within-domain edges. It does not establish a causal developmental architecture, a validated vulnerability score, or reduced inter-domain integration by a formal normalized graph metric.

### 4.1 The architecture of vulnerability: from systemic fragility to structural dependency

The central observation was higher global strength in the ASD network. This is a summary of absolute edge weights, not network density or direct evidence of structural rigidity. We therefore interpret the pattern cautiously as potentially consistent with concentrated associations among selected developmental abilities. Whether this pattern produces reduced resilience or compensatory capacity requires longitudinal and intervention-based evidence.

The observed network organization may provide a behavioral-level hypothesis relevant to theories of altered integration in ASD, but it should not be equated with neural connectivity. RL was the highest-strength node in the ASD network, whereas COM had the highest strength in the TD network. This difference identifies candidate nodes for further study; it does not establish that RL is a unique bottleneck or that altering it would have system-wide consequences.

### 4.2 Hypothesis generation: from static association to potential structural dependencies

The cross-sectional design prevents causal or developmental interpretation of the DAG (31, 32). Although bootstrap support was used to retain stable edges, it does not resolve Markov equivalence or establish temporal precedence. We therefore present the directed structures as conditional-dependence hypotheses for longitudinal testing, rather than as evidence for bottom-up or top-down developmental cascades.

Any account in which symptoms actively inhibit foundational skills remains speculative in the present dataset. Such mechanisms require longitudinal designs, explicit temporal modeling, and ideally intervention or quasi-experimental evidence.

The sex-stratified findings should be interpreted cautiously. The ASD female subgroup was much smaller than the male subgroup, and no sample-size-balanced sensitivity analysis was performed. Consequently, the observed differences in centrality and edge patterns are descriptive and should be replicated before they are interpreted as sex-specific developmental pathways, in line with prior work showing that gender-specific pathways derived from network and Bayesian-network models can vary substantially across settings (33).

If replicated in longitudinal and independent cohorts, the present framework may help prioritize integrated monitoring of receptive language and communication alongside adaptive functioning. It does not yet support selecting interventions on the basis of network position alone. Instead, its clinical value is currently hypothesis-generating and may guide future studies of whether coordinated, cross-domain supports improve developmental outcomes.

### 4.3 Limitations and future directions

This study has several limitations. First, its cross-sectional design prevents causal interpretation of the DAG orientations and cannot resolve statistically equivalent graph structures. Second, structural vulnerability was an interpretive descriptor, not a validated scalar, and we did not estimate a normalized formal between-group metric of inter-domain integration. Third, centrality stability was evaluated within the EBICglasso specification only; alternative estimators, tuning parameters, and node sets were not examined. Fourth, edge-level NCT p values were unadjusted and should be considered exploratory. Fifth, we did not estimate the proportion of adaptive-functioning variance uniquely explained by RL relative to COM, SOC, or DLY; network centrality should not be interpreted as unique explained variance. Sixth, age was not adjusted for and no separately modeled developmental quotient was included; other potential confounders, including unmeasured clinical, socioeconomic, and contextual characteristics, may also have influenced the observed associations. Seventh, the ASD female subgroup was smaller than the male subgroup and was not examined with balanced-sample sensitivity analyses. Eighth, the selected node set did not include executive functioning, sensory processing, or play skills, which could affect the estimated topology and centrality ordering. Finally, the model has not been tested in an independent ASD cohort. Longitudinal, covariate-adjusted, adequately powered, and externally replicated studies are required to evaluate the proposed model (34).

## 5 Conclusion

In conclusion, this study describes a symptom-ability network configuration in a large ASD sample and identifies RL and COM as candidate central and bridging nodes. The Structural Vulnerability Model should be regarded as a hypothesis-generating framework. Its developmental, clinical, and intervention implications require confirmation in longitudinal, independently replicated, and sensitivity-tested studies.

## Data Availability

The data supporting the findings of this study are available on the Autism Center of Excellence l aboratory’s GitHub page at https://github.com/ACE-UCSD/Autism-Sex-Differences-Analysis-Pathway. Analytic R scripts used to estimate the reported models are available from the corresponding author upon reasonable request.
